# Evaluation of Post-Surgical Recovery in Olive Flounder (*Paralichthys olivaceus*) by Assessing Behavior, Heart Rate, and Wound Healing

**DOI:** 10.3390/ani14030363

**Published:** 2024-01-23

**Authors:** Myungsung Koo, Man-Ki Jeong, Inyeong Kwon

**Affiliations:** 1 Division of Fisheries Engineering, National Institute Fisheries Science, Busan 46083, Republic of Korea; kms2736@korea.kr; 2 Department of Smart Fisheries Resource Management, Chonnam National University, Yeosu 59626, Republic of Korea; jmgdeux@jnu.ac.kr

**Keywords:** recovery, bio-logger, post-surgery

## Abstract

**Simple Summary:**

This study assessed behavior, heart rate, and wound healing in flounder to evaluate the appropriate recovery period after surgical implantation of a bio-logger. Compared to the non-operated fish, the operated fish exhibited stable levels after the 3rd 4th day in Experiment 1 (22 °C). Statistical analyses based on heart rate in Experiment 1 indicated that the appropriate post-surgery recovery time point was approximately 3 days, which represents the point at which behavioral fluctuations stabilized. In the case of Experiment 2 (28 °C), abnormal behavioral patterns (e.g., tilted swimming) and changes in average swimming time and daily heart rate were found to stabilize after 4 days post-surgery.

**Abstract:**

This study examined the post-surgery recovery of olive flounder (*Paralichthys olivaceus*) following tag insertion by analyzing behavior, heart rate, and wound healing. The experiments used 30 individuals (length: 38.67 ± 2.12 cm; weight: 742.48 ± 116.41 g). Heart rate was measured using a DST milli-HRT (Star-Oddi) bio-logger. To assess the influence of water temperature on the recovery process after surgical tag insertion, behavioral analyses, heart rate, and wound healing were conducted in two experimental groups: Experiment 1 (22 °C, optimal water temperature); Experiment 2 (28 °C, high water temperature); and control group (22 °C, non-operated fish). The experiment was repeated twice over a 7-day period for each experimental group. Compared to the non-operated fish, the operated fish exhibited stable levels after the 3rd to 4th day in Experiment 1. Statistical analyses based on heart rate in Experiment 1 indicated that the appropriate post-surgery recovery time point was approximately 3 days, representing the point at which behavioral fluctuations stabilized. In the case of Experiment 2, abnormal behavioral patterns (e.g., tilted swimming) and changes in average swimming time and daily heart rate were found to stabilize after 4 days post-surgery.

## 1. Introduction

Tags are a tool that is being increasingly used to study the movement of aquatic organisms in natural and aquaculture environments through telemetry [1]. Telemetry is the tagged, passive spatial, and temporal monitoring of movement and habitat use of marine and freshwater species. This technique has revolutionized aquatic animal tracking by providing insights into their distribution and survival [2]. Notably, the tags are compact and have data storage capabilities, efficient flash memory, satellite sensing capabilities, extended battery life, and telemetry tags that deliver environmental, behavioral, and physiological data in real-time. This provides more information about individual animal behavior than ever before. More importantly, these devices are easily accessible [2,3] when they are attached externally [4,5,6,7,8,9] or inserted internally [6,8,10,11,12,13,14]. Historically, when tags are attached externally, problems such as loss of the tag or increased risk of predation arise; therefore, the tag is often inserted internally through a surgical operation [6,8,10,11,12,13,14,15]. Fish undergoing surgery for internally inserted tags, however, can become stressed during handling, anesthesia, incision and suturing, and recovery [1,3,15].

From a statistical standpoint, it is crucial to insert as many tags into as many fish as possible to ensure that behavioral and physiological data derived from experimental fish are representative of the target fish population. However, due to regulations on the ethics of animal experimentation, especially the 3R (replacement, reduction, and refinement) guidelines, researchers are encouraged to refrain from using animals if alternative methods are available. Moreover, it is recommended to minimize the number of animals used in experiments and improve methods to reduce animals’ pain [3,16]. Therefore, tag experiments targeting fish must reduce the number of animals included while minimizing stress during the experiment. In animal monitoring, ensuring that the observed animals are representative of the population is essential [15]. Hence, when using telemetry fish behavior and physiology should not change after compared to before tagging [15]. Thus, researchers using tags to observe fish behavior do not use data obtained from surgical procedures until the fish recovers (i.e., before the fish’s behavior returns to a normal state) [3].

Based on the aforementioned considerations, numerous studies have quantified the recovery period of fish to establish reliability standards for data obtained from internally inserted tags [1,12,15,17,18,19,20,21,22,23,24,25]. The criteria for determining the degree of recovery after surgical tag insertion include: the degree of healing of the surgical area (i.e., infection of the affected area, etc.) [19,21,22,24]; growth [1,25]; survival (mortality) [1,17,19,25]; feeding behavior [17]; body condition [1,18]; metabolic rate [18]; heart rate stability [12,13,15]; and swimming behavior [15,18,19,20,21].

Nevertheless, most studies evaluating recovery after tag insertion have focused on spindle-shaped and lateral-shaped pelagic fish swimming in the upper and middle layers of the water column [1,11,12,15,18,19,20,21,22]. Few studies have been conducted on benthic fish species such as flatfish [17,25]. Moser et al. [17] observed the degree of influence of the fishes after surgery through survival and feeding behavior after transmitter insertion surgery in adult English soles (*Pleuronectes vetulus*). Fabrizio and Pessutti [25] compared the survival and growth of two fish species (black sea bass and summer flounder) according to their degree of exposure to clove oil, to evaluate recovery after surgery. In particular, recent studies with olive flounder (*Paralichthys olivaceus*)—a highly valued farmed fish in Korea—have focused on inserting tags into the body to quantify health information (e.g., stress-related endpoints) of the fish as part of the “smartization” of aquaculture systems [14]. However, very few studies have examined the impact of these tools on olive flounder or on the recovery process that follows surgery. Furthermore, since the unique farming environment of the flounder in Korea (mostly flowing water) is greatly affected by water temperature, tagging experiments in actual fish farms are deemed necessary. Additionally, due to their nature, ectothermic animals undergo physiological and chemical changes depending on the surrounding environment [26]. Water temperature is a major factor that directly affects physiological functions, such as the metabolism of fish [27]. The degree of wound healing in fish varies due to metabolic changes influenced by water temperature [28]. Therefore, before implementing a tagging system, it is crucial to quantify the influence of water temperature on the degree of recovery. Therefore, our study characterized the degree of recovery at the surgical area, abnormal behavior, and heart rate to determine the degree and duration of recovery after tag insertion in olive flounder. Additionally, as mentioned earlier, we compared and analyzed the influence of water temperature on the recovery of flounder.

## 2. Materials and Methods

### 2.1. Experimental Condition, Research Ethics and Procedures

For the experiments, flounders cultured in land tanks in the Yeosu area were transported to the laboratory and acclimatized at 22 °C ± 0.5 °C for two weeks prior to conducting the experiments. Flounders used in the experiment comprised 30 individuals with a length of 38.67 ± 2.12 cm and a weight of 742.48 ± 116.41 g. Length and weight were measured during tag insertion surgery two weeks after acclimation. The water tank (2 × 1 × 1.2 m) used in the experiment included a lab-scale circulating filtration water tank (2000 Electronics, Busan, Republic of Korea), a dissolved oxygen generator (LP-80A; Youngnam Electronics, Busan, Republic of Korea), a cooler (DA-2000-LW; Daeil, Busan, Republic of Korea), three heaters (TLW-A50-2kW; Dongwha Electronics, Busan, Republic of Korea), and a skimmer (SL-2000; 21st Century Hi-Tech Co., Ltd., Daejeon, Republic of Korea).

To determine the influence of temperature on the degree of recovery after tag insertion, two experiments were conducted after surgery to compare changes in behavior: Experiment 1 (22 °C, optimal water temperature), Experiment 2 (28 °C, high water temperature), and control (22 °C, non-operated fish). In particular, in the control group, to compare the swimming behavior of flounders before and after surgery, an experiment was conducted under the same water temperature conditions as in Experiment 1, which is the optimal water temperature range (22 °C) for the growth and activity of flounders. Experiments for each of the three experimental groups were repeated twice, and the overall experiment was conducted for 7 days using five flounders per experimental group.

For the experimental conditions, dissolved oxygen was maintained at 9–10 mg/L and salinity at 32–33 psu in all experimental plots. Additionally, the artificial lighting intensity (9–10 lx) was fixed, to allow only water temperature to be considered as a variable for postoperative recovery. The photoperiod remained the same for 24 h. Regarding feeding, 1% of the fish’s body weight was provided once a day during the acclimatization and experiment period every morning, and approximately 20% of the water was exchanged daily using water from a tank maintained at the same water temperature as the experimental water. Animal care and all experimental procedures adhered to national rules and regulations. Ethical permission was granted by the Animal Research Ethics Committee of Chonnam National University (CNUIACUC-YS-2023-12).

To implant bio-loggers (DST milli-HRT, Star-Oddi, Gardabaer, Iceland) for measuring heart rate, the fish that had been acclimatized for two weeks in an aquarium were injected with 100 mg/L ethyl-3-aminobenzoate methanesulfonate (MS222). The surgery was performed after anesthesia for 3–4 min in seawater diluted with an anesthetic (Sigma-Aldrich Inc., St. Louis, MO, USA). The surgery was completed in approximately 5 min, in consideration of the stress experienced by the flounder and the time needed for the anesthesia to wear off. An incision of approximately 1.5–2 cm was made near the heart, and the affected area was sutured. At the end of the experiment, a live subject was anesthetized and data were retrieved from the logger. The logger settings were configured to measure heart rate at 10-min intervals, and ECG data were measured at 3-h intervals to confirm the validity of the data. The frequency was set to 100 Hz; this is a standard setting for measuring fish heart rate.

### 2.2. Data Analysis

Heart rate stability, behavior, and wound healing were monitored to assess the post-surgery recovery of olive flounder. In this study, normal and abnormal behavior types were differentiated through a preliminary literature review on the swimming behavior of flounders, as illustrated in Table 1. Based on this standard, the behavior in the recorded video was analyzed using the naked eye and the EthoVision XT program version 17. Additionally, abnormal behavior types, the general movement of flounders (e.g., swimming off the bottom or moving attached to the bottom), and swimming time were analyzed during the experiment. The degree of wound healing was determined following the criteria outlined by Lopes et al. [23] to compare the post-surgical conditions of flounders (Table 2).

In the case of flounders’ heart rate, data preprocessing was conducted in two steps. Initially, the data from the logger were converted to Mercury v. data and were collected using the 6.14 software and the associated Communication Box (Star-Oddi, Gardabaer, Iceland). In the first stage, the verification quality index (QI), which automatically assesses the data quality in the logger, was employed to determine the quality of data; this ranged from 0 to 3 (QI0 = Great, QI1 = Good, QI2 = Fair, and QI3 = Poor). Only data from QI0 and QI1 were utilized. In the second stage, ECG data were used to establish standards for the maximum and minimum data values, and the HRT Analyzer 1.0.2 (Star-Oddi; Gardabaer, Iceland) was employed for the analysis. Additionally, statistical analysis was conducted to ascertain the significance of the results in terms of behavior, wound healing, and changes in heart rate over time. For behavioral analysis and heart rate changes over time, a one-way ANOVA test was performed to compare three or more groups. For wound healing, a *t*-test was employed for pairwise comparisons. A *p*-value < 0.05 was considered statistically significant.

## 3. Results

### 3.1. Behavior Monitoring

After analyzing the behavior of the unoperated flounder over 7 days, the average swimming time and swimming frequency (when leaving the bottom and moving while attached to the bottom) were found to be 9.06 s, 5.78 instances for swimming off the bottom, and 7.45 instances of moving while attached to the floor, respectively (Table 3). Examining the frequency of behavior changes within the normal water temperature range (Experiment 1) in the operated flounder (refer to Table 4) revealed that the swimming time was observed to be less than 9.01 s from the 3rd day after surgery compared to the unoperated flounder (*p* > 0.05). Similarly, the average number of instances of swimming off the bottom was fewer than five times, starting from the 4th day after surgery. Moreover, cases of moving while stuck to the floor were below the average value for the operated flounder 4 days post-surgery. In the high-temperature experiment (Experiment 2), swimming time decreased to less than 10 s as time passed, compared to days 1 and 2, and the frequency of swimming off the bottom also decreased; however, it remained higher overall compared to the unoperated flounder. Upon examining the criteria for flounder behavior discrimination outlined in Table 2, the head-up type (C) exhibited abnormal behavior. Particularly in comparison to other experimental groups, abnormal behavior was more frequent until the 3rd day in Experiment 2 (*p* < 0.05, Table 5).

### 3.2. Degree of Wound Healing

**Figure 1:**
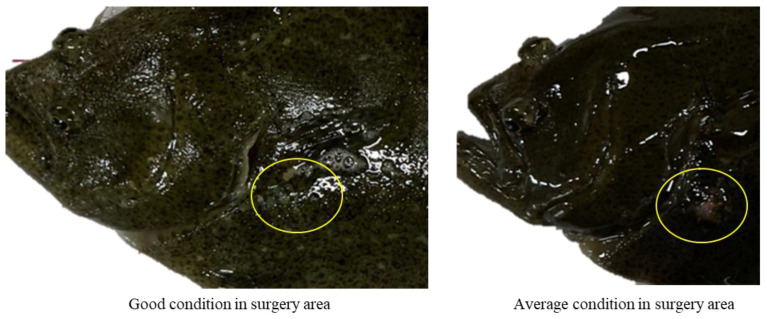
Wound healing after surgical operation in flounders.

As shown in Table 6 and Figure 1, most of the surgical sites of the flatfish were maintained in good condition. However, in Experiment 2, one individual had no signs of infection but was deemed to be in average condition; this was because it exhibited incomplete wound closure (*p* > 0.05).

### 3.3. Heart Rate

**Figure 2:**
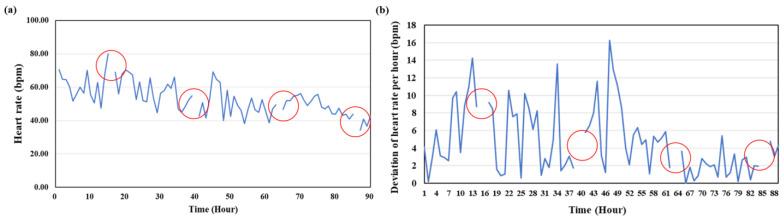
Heart rate change of the flounder after surgery in Experiment 1. (**a**) Heart rate variations over the course of 90 h post-surgery. (**b**) Deviation of heart rate change per hour after surgery. The red circle indicates the heart rate at feeding time excluded from the analysis.

Upon analyzing heart rate changes in the flounder post-surgery, in Experiment 1, the heart rate showed a gradual decrease over time, as depicted in Figure 2a. Specifically, as observed in Figure 2b, the flounder’s heart rate changed on an hourly basis, with variations appearing to attenuate after approximately 52 h (*p* < 0.05). In other words, at approximately 3 d post-surgery, the change (deviation) in heart rate per hour seemed to exhibit a gradually stabilizing pattern (*p* < 0.05). However, it is worth noting that the heart rate during feeding time was excluded from the analysis, and the heart rate range in this segment was 61.9–73 bpm, which was consistently high regardless of the passage of time.

**Figure 3:**
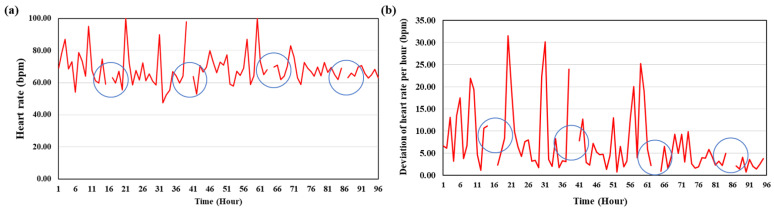
Heart rate change of the flounder after surgery in Experiment 2. (**a**) Heart rate variations over the course of 96 h post-surgery. (**b**) Deviation of heart rate change per hour after surgery. The blue circle indicates the heart rate at feeding time excluded from the analysis.

After analyzing the change in heart rate of flatfish post-surgery in Experiment 2, it was found that the heart rate tended to increase and decrease repeatedly over time, as depicted in (a) (*p* > 0.05) and (b) of Figure 3. Notably, the deviation of the flounder’s heart rate, which varied per hour, decreased after approximately 64 h. In other words, at approximately 3 d post-surgery, the change (deviation) in heart rate per hour seemed to gradually exhibit a stable pattern (*p* < 0.05). However, the heart rate during feeding time was excluded from the analysis and in this segment, the heart rate range was 66.3–71.57 bpm; this is consistently high and is regardless of the passage of time. The change in heart rate over the 7 days of the experiment showed an average difference of about 19.29 bpm between Experiments 1 and 2 (Table 7). The heart rate of the flatfish appeared to stabilize after about 3 d in Experiment 1 and after 4 d in Experiment 2 (*p* > 0.05).

## 4. Discussion

In this study, experiments were conducted to monitor changes in behavior, surgical area recovery, and heart rate stabilization, and to examine the time required for the flounder to recover after surgery. Due to a lack of standard behavior criteria, the types of behavior between non-operated and operated individuals were compared.

The criteria for recovery of activity used in this study were based on the frequency of swimming and swimming time. When comparing the average number of swimming instances and speeds of unoperated flounders, the values stabilized after the 3rd to 4th day in Experiment 1. Based on the heart rate in Experiment 1, our statistical analysis demonstrated that the appropriate time point for post-surgery recovery was approximately 3 d, when any changes in behavioral output became stable. In the case of Experiment 2, the abnormal behavior pattern (C), and the deviation of average swimming time and daily heart rate were found to stabilize 4 d after surgery, but the pattern of continuous movement while landing on the floor did not decrease.

In examining the literature on flounder behavior (Table 1), it is difficult to distinguish abnormal behavior based on simple movements while attached to the bottom. The fact that there was more movement in Experiment 2 compared to Experiment 1 suggests a tendency to rest or seek the appropriate water temperature range. In the case of swimming off the bottom, despite differences in the size of the fish used in this experiment, as well as methodological differences, our findings mirrored those of another study that compared the swimming time of smaller flatfish (4 g) infected with *Neoheterobothrium hirame* with that of their uninfected counterparts. In particular, the authors reported that when flatfish are unhealthy, they swim off the bottom for a longer period of time [34].

Føre et al. [15] determined the postoperative recovery of Atlantic salmon based on sensor-based heart rate and behavior. Heart rate recovery was found to require a minimum of 1.27 d and a maximum of 5.75 d, with slight differences depending on individual characteristics among the nine fish [15]. Brijs et al. [11] reported that when recovery was evaluated based on heart rate changes by inserting a bio-logger into rainbow trout (*Oncorhynchus mykiss*), the average daily heart rate tended to decrease significantly after 4 days.

Additionally, Brijs et al. [12] reported that when evaluating the heart rate stabilization time after logger insertion in 11 rainbow trout, it stabilized at 42 bpm after 72 h. Although the fish species analyzed in this study differed from those in the aforementioned study, the authors found that 4 d recovery time was appropriate after the surgical logger insertion. Moreover, in Experiments 1 and 2, there was a difference in the standard stabilizing heart rate, suggesting that the electrical stimulation that initiates and controls the speed and rhythm of heart contraction in fish is affected by temperature [35].

Previous studies have also reported that the wound-healing process varies depending on the tag size, incision size, suture spacing, surgical period, and surgery time [21]. In addition, it has been reported that water temperature and food nutrients affect the degree of wound healing in fish [28]. In this experiment, only one flounder in Experiment 2 exhibited an average condition of the surgically affected area (*p* > 0.05), whereas the rest showed a good condition for 7 d after surgery. In particular, the surgical period, determined by Lopes et al. [23] to be appropriate at 6 min or less, was adhered to in most cases (5 min or less) when there was an incision size of approximately 2 cm. The surgery was performed with an incision of approximately 1.5–2 cm. Jensen et al. [28] determined the degree of healing of wounds caused by contact within the cage of Atlantic salmon (*Salmo salar*) under different water temperatures (4 °C or 12 °C) and three different diets (one control diet [Control], a diet with additional organic zinc [Zn], and a diet with a mix of additives [Zn+]). Wound healing was faster under conditions of high water temperature (12 °C), and in the case of food, fish that ate food containing Zn healed faster [28]. However, in this experiment, the degree of wound healing of flatfish was determined based on water temperature conditions, and there was no statistically significant difference in the degree of wound healing in Experiments 1 and 2 (*p* > 0.05). This is because changes in metabolism and immunity have a greater impact at lower than optimal water temperatures for fish [28,36,37]. Upon comparison with the optimal water temperature range for flounders, Experiment 2 showed good wound healing under high water temperature conditions.

## 5. Conclusions

In this study, post-surgery healing in flatfish after tag implantation was assessed based on behavior, wound healing, and heart rate stability under two different water temperatures. In the initial stages post-surgery, swimming behavior and heart rate remained generally high, and a discernible pattern of stabilization emerged over a specific period. Notably, within the optimal water temperature range, heart rate and behavior frequency typically stabilized 3–4 d after surgery. However, in the experiments conducted under higher water temperatures, recovery tended to occur slower than in those in which the water temperature range was optimal.

## Figures and Tables

**Table 1:** Criteria for assessing flounder behavior.

Code	Classification of Abnormal Types	Explanation	Causes	References
A	Normal behavior	Remaining in the same space, firmly positioned on the floor without movement		NIA [29] Kawabe et al. [30]
B		Swim-glide-land (Burial)	Normal swimming Feeding behavior	Kawabe et al. [30]
C	Tilted swimming	Swimming at an angle or with the head held vertically	Deformity of the vertebrae Bladder dysfunction Water quality issues Internal parasites or infections Behavioral changes or stress	Webb [31] Sirri [32]
D	Raising the mouth	Gaping at the surface of the water Maintaining a horizontal angle of 15 degrees or more for 5 s on the water surface	Decreased dissolved oxygen	NIA [29]
E	Slow swimming at the water surface	Floating slowly on the surface of the water Horizontal angle less than 15 degrees from the water surface, stop for more than 10 s	Infections and metabolic diseases Changes in water temperature	NIA [29] NIFS [33]
F	Turning	Turning the body from the bottom of the tank to the water surface at high speed Body rotation and rising more than once	Disease infection or change in water quality	NIA [29]
G	Rubbing	The act of quickly rubbing the body by repeatedly turning over, followed by adopting an upright position once more Turning over and fluttering repeatedly more than once	Parasitic/bacterial infections	NIA [29]

**Table 2:** Determination of recovery criteria (source: Lopes et al. [23]).

Postsurgical Ranking	Description
Good	Incision closed
	All sutures retained (applies to one week post-surgery only)
	No signs of infection
Average	Incision not fully closed
	Most sutures retained (applies to one week post-surgery only)
	No signs of infection
Poor	Large incision opening
	Most sutures lost (applies to one week post-surgery only)
	Signs of infection and necrotic tissue around the incision site

**Table 3:** Results of behavioral analysis of flounders (Control).

Time (d)	Average Swimming Time (s)	Average Swimming Frequency (Off the Bottom)	Average Swimming Frequency (When Attached to the Bottom)	Abnormal Behavior (Type-Frequency)
1	8.59 ± 5.14	5.88 ± 5.63	9.5 ± 4.12	-
2	8.20 ± 4.23	5.67 ± 5.41	7.9 ± 5.62	C-1
3	8.62 ± 4.75	6.08 ± 5.69	7.1 ± 7.72	-
4	8.25 ± 5.12	5.58 ± 4.33	9.7 ± 4.33	-
5	9.79 ± 5.22	5.42 ± 4.12	6.0 ± 5.20	-
6	10.00 ± 5.84	6.00 ± 5.98	6.7 ± 4.13	-
7	9.96 ± 4.60	5.83 ± 4.13	5.2 ± 5.21	-
Average	9.06 ± 4.99	5.78 ± 5.04	7.45 ± 5.19	

**Table 4:** Results of behavioral analysis of flounders (Experiment 1).

Time (d)	Average Swimming Time (s)	Average Swimming Frequency (Off the Bottom)	Average Swimming Frequency (When Attached to the Bottom)	Abnormal Behavior (Type-Frequency)
1	15.90 ± 7.87	13.50 ± 7.15	7.00 ± 3.53	C-2
2	11.63 ± 7.28	14.00 ± 8.88	9.13 ± 6.91	C-1
3	9.01 ± 4.48	11.79 ± 7.88	14.50 ± 7.12	-
4	8.65 ± 5.65	5.23 ± 5.31	6.10 ± 7.04	-
5	8.00 ± 4.39	5.21 ± 4.04	7.00 ± 7.64	-
6	7.93 ± 4.63	5.33 ± 5.24	5.71 ± 5.60	-
7	7.96 ± 4.79	5.04 ± 4.80	5.50 ± 5.53	-
Average	9.99 ± 5.58	9.02 ± 6.19	8.45 ± 6.19	

**Table 5:** Results of behavioral analysis of flounders (Experiment 2).

Time (d)	Average Swimming Time (s)	Average Swimming Frequency (Off the Bottom)	Average Swimming Frequency (When Attached to the Bottom)	Abnormal Behavior (Type-Frequency)
1	14.45 ± 13.62	9.67 ± 7.50	12.78 ± 8.77	C-10
2	25.08 ± 16.65	11.75 ± 5.60	18.42 ± 12.10	C-5
3	9.21 ± 5.15	10.42 ± 6.95	24.88 ± 13.99	C-10
4	6.47 ± 1.80	8.92 ± 4.93	26.75 ± 11.01	C-1
5	6.68 ± 1.61	8.25 ± 4.41	25.54 ± 11.57	-
6	7.05 ± 1.78	9.17 ± 6.06	22.38 ± 15.56	-
7	6.81 ± 1.83	7.83 ± 4.97	24.04 ± 16.44	-
Average	10.82 ± 6.06	9.43 ± 5.77	22.11 ± 12.78	

**Table 6:** Post-surgery wound healing in olive flounders.

	Experiment 1	Experiment 2
Good	10	9
Average	-	1
Poor	0	0

**Table 7:** Post-surgery average heart rate changing of olive flounders in Experiment 1 and 2.

Time (d)	Experiment 1 (bpm)	Experiment 2 (bpm)
1	61.03 ± 8.48	67.54 ± 11.86
2	52.41 ± 8.98	70.14 ± 11.88
3	50.0 ± 4.3	68.07 ± 5.35
4	43.64 ± 6.38	71.08 ± 5.95
5	46.20 ± 6.66	66.08 ± 9.16
6	43.27 ± 8.26	68.43 ± 7.61
7	46.86 ± 7.73	67.10 ± 8.24

## Data Availability

All data are stated in the manuscript.
